# Localized Darier’s disease with a blaschkoid distribution in a pregnant woman

**DOI:** 10.1016/j.jdcr.2025.01.031

**Published:** 2025-02-25

**Authors:** Nina Patel, Florencia Anatelli, Christina Kranc

**Affiliations:** a Stritch School of Medicine, Loyola University Chicago, Maywood, Illinois; b Southwest Skin Specialists, Phoenix, Arizona

**Keywords:** blaschkoid, Darier’s disease, genetic mosaicism, pregnancy

## Introduction

Darier’s disease, also known as keratosis follicularis or Darier-White disease, is an autosomal dominant disease due to ATP2A2 gene mutation, which encodes the Ca^2+^ pump in the endoplasmic reticulum, SERCA2.^1^ The condition affects approximately 1 in 30,000 to 1 in 100,000 individuals, with high penetrance but variable expressivity.^2^, ^3^, ^4^ The disease often presents in adolescence and coincides with female reproductive age, though men and women are affected equally. Here, we present a rare case of focal Darier’s disease with a blaschkoid distribution in a pregnant patient with no peripheral involvement.

## Case report

**Fig 1:**
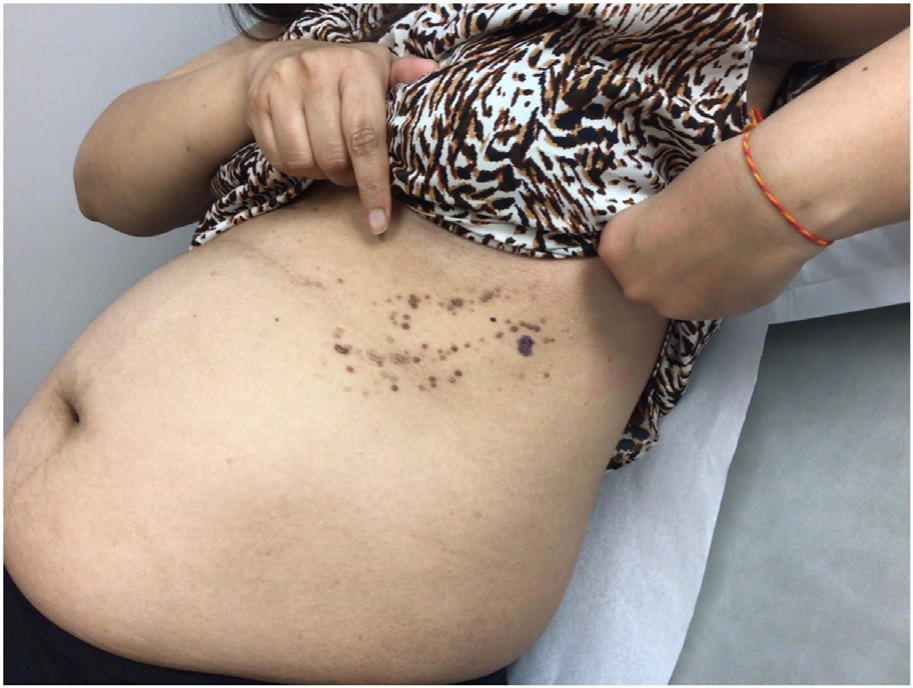
Multiple clusters of hyperkeratotic papules in a blaschkoid distribution on the left rib cage on first patient encounter.

A 36-year-old G1P0 pregnant woman (20 weeks gestation) of Asian descent presented with a 4-month history of scaly skin lesions on the left trunk. The eruption of pruritic and inflamed lesions started on her left side and spread to the upper abdomen. The rash appeared around the time the patient experienced viral infection symptoms, including a cough. Review of systems was otherwise negative. She denied a known personal and family history of skin disease. Physical examination revealed multiple clusters of hyperkeratotic, verrucous 2-4 mm papules on the left rib cage, extending inferomedially to her left upper abdomen in a linear/dermatomal distribution as shown in Fig 1. No nail, mucosal, or groin lesions were noted.

**Fig 2:**
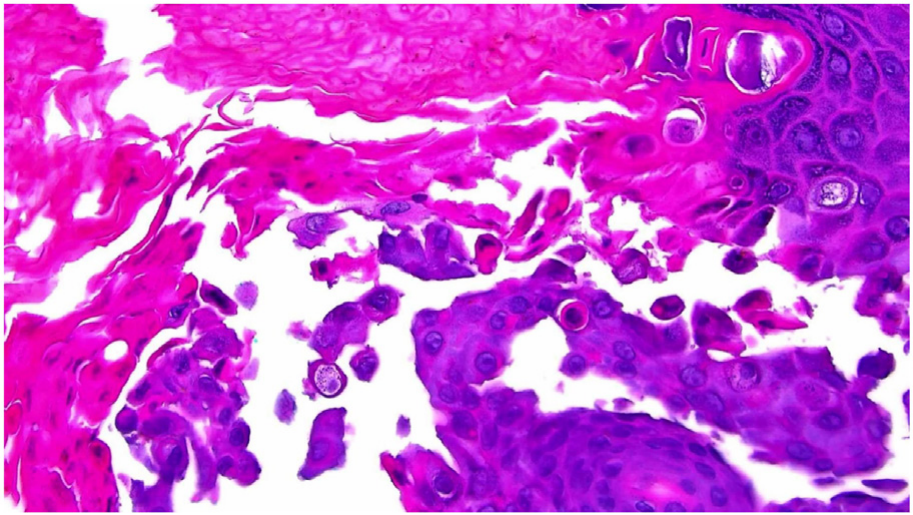
High-power histologic image depicting foci of suprabasal cleft with acantholytic cells, corps ronds, and grains within the granular zone (Magnification 40×).

Shave biopsy was performed on the left rib cage. Histopathologic examination revealed mild epidermal acanthosis and papillomatosis, with foci of suprabasal clefts containing acantholytic cells in the granular zone and cornified layer, as well as hyperkeratosis and parakeratosis. Additionally, dyskeratosis with corps ronds and grains within the granular zone is reflected in Fig 2. Histologic findings with this clinical picture were most consistent with a diagnosis of localized Darier’s disease.

Topical triamcinolone acetonide 0.1% cream was prescribed for actively inflamed lesions. She was advised to avoid friction and keep the skin cool and dry. Many of the lesions cleared after 4 weeks without new lesions.

## Discussion

Clinically, Darier’s disease presents as pruritic, greasy, keratotic, and often disfiguring plaques in seborrheic and flexural regions. Lesions often fluctuate and exacerbations have been reported from environmental exposures.^4^ Patients are highly susceptible to secondary viral and bacterial skin infections that require surveillance, prevention, and treatment.

In 1906, Kreibich first identified the localized pattern of Darier’s disease where only involved skin possessed ATP2A2 mutations.^5^ Darier’s has since been defined by 2 variations: type 1, where lesions follow focal Blaschko’s lines unilaterally on unaffected skin (composing roughly 10% of all cases) and type 2, characterized by focal areas of increased severity on a background of generalized Darier’s disease.^4^ Blaschko’s lines reflect the disease’s mosaic nature in ectodermal development where skin involvement is circumscribed, patchy, or in a linear arrangement.^4^ Researchers postulate the phenotypic expression of mosaicism from epidermal keratinocytes could indicate loss of heterozygosity resulting from postzygotic somatic mutations along Blaschko’s lines.^5^

Given the linear presentation of lesions, a clinical differential diagnosis to consider is inflammatory linear verrucous epidermal nevus syndrome which can present similarly to focal Darier’s disease with verrucous papules following Blaschko’s lines. linear verrucous epidermal nevus syndrome can be distinguished by earlier onset and lack of spontaneous regression, however histopathologic analysis is diagnostic.

Histologic differential diagnosis should include other acantholytic dermatoses including familial benign pemphigus (Hailey–Hailey disease) and transient acantholytic dermatosis (Grover’s disease). Hailey–Hailey disease is differentiated by biopsy, but Grover’s and Darier’s are histologically identical. Darier’s is distinguished from Grover’s through clinical presentation, though differentiation is challenging since both can present with blaschkoid appearance. Grover’s disease is significantly more common in males with a prevalence ratio of up to 7:1 and has been documented predominantly in white individuals.^6^ Additionally, Grover’s disease typically arises in middle-aged to older adults with presentation often around the fifth to seventh decade of life.^6^ In this 36-year-old, Asian woman, a diagnosis of Grover’s disease was less favored as a result. A family history of Darier’s can be helpful for diagnosis, but may not be identifiable due to variable expressivity. Genetic testing is diagnostic. The patient did not receive genetic testing as she declined due to personal preferences.

Symptomatic management is key. Avoidance of precipitating factors (ie sun exposure), sunscreen use, and proper hygiene practices are vital. Severe disease is treated with oral and/or topical retinoids which have a reported 90% efficacy rate.^2^ Case reports also indicated efficacy with topical 5-fluorouracil. However, this is highly contraindicated in pregnancy due to retinoids’ teratogenic properties. With lesser efficacy, topical steroids can be safely utilized in pregnancy.^7^

This is one of a few reports to document Darier’s disease in pregnancy and is the first report of localized blaschkoid Darier’s disease in a pregnant patient. The disease aggravations during pregnancy or menses, and onset near puberty could be evidence of the hormonal dependence of Darier’s disease.^8^ One study found relative estrogen excess ameliorates keratosis follicularis and estrogen-dominant oral contraceptives markedly improved patients’ condition.^8^ Our patient case supports this theory as she presented while pregnant for onset of her rash.

Darier's disease can complicate obstetric care. Traumatic vaginal birth may be exacerbated by reduced skin elasticity, while cesarean sections carry a risk of scarring and infection.^9^ Infection on maternal skin can also pose a threat for neonatal sepsis. Additional concerns include folliculitis and the inability to breastfeed if breast lesions are present.

Darier’s disease exhibits autosomal dominant inheritance, therefore there is a 50% chance that offsprings are affected with variable penetrance; the disease carries a phenotypic spectrum from mild to severe forms that could impact quality of life. Patients with Darier’s disease are encouraged to seek prenatal counseling. Mutations in the ATP2A2 gene lead to the ‘classic’ form of Darier’s disease. More recent genetic studies have confirmed that individuals with localized Darier’s disease are mosaics for this mutation. The risk of transmission of generalized Darier’s disease will depend on whether the germline is affected.^5^ In our case, the inheritance (germline vs somatic) remains unclear, making the choice of prenatal counseling challenging from the provider perspective.

It is important for dermatologists to recognize mosaic genodermatoses in practice, due to potential transmission to offspring.^10^ Cutaneous mosaicism is easily visualized compared to other organ systems, as affected tissue typically follows patterns, like the lines of Blaschko.^10^ However, cutaneous disease burden cannot be directly correlated with germline transmission risk. Further studies are warranted to understand the genetic mosaicism of Darier’s disease and how this presentation reflects a de novo postzygotic somatic mutation in heterozygous state. This is the first documented case of localized Darier’s disease in a pregnant patient and demonstrates that a diagnosis of Darier’s disease should be considered for pregnant women presenting with a focal pruritic blaschkoid rash with little peripheral involvement.

## Conflicts of interest

None disclosed.
